# Heterogeneous associations of gut microbiota with Crohn’s disease activity

**DOI:** 10.1080/19490976.2023.2292239

**Published:** 2023-12-17

**Authors:** Susanne Pinto, Elisa Benincà, Gianluca Galazzo, Daisy Jonkers, John Penders, Johannes A. Bogaards

**Affiliations:** a Biomedical Data Sciences, Leiden University Medical Center, Leiden, Netherlands; b Centre for Infectious Disease Control, National Institute for Public Health and the Environment (RIVM), Bilthoven, Netherlands; c School for Nutrition and Translational Research in Metabolism (NUTRIM), Maastricht University, Maastricht, Netherlands; d Department of Medical Microbiology, Infectious Diseases and Infection Prevention, Maastricht UMC, Maastricht, Netherlands; e Department of Gastroenterology-Hepatology, Maastricht UMC, Maastricht, Netherlands; f Epidemiology and Data Science, Amsterdam UMC location Vrije Universiteit Amsterdam, Amsterdam, Netherlands; g Institute for Infection and Immunity (AII), Amsterdam UMC, Amsterdam, Netherlands

**Keywords:** Microbiota, Crohn’s disease, disease activity, quantile regression, microbial ecology

## Abstract

The multi-factorial involvement of gut microbiota with Crohn’s disease (CD) necessitates robust analysis to uncover possible associations with particular microbes. CD has been linked to specific bacteria, but reported associations vary widely across studies. This inconsistency may result from heterogeneous associations across individual patients, resulting in no apparent or only weak relationships with the means of bacterial abundances. We investigated the relationship between bacterial relative abundances and disease activity in a longitudinal cohort of CD patients (*n* = 57) and healthy controls (*n* = 15). We applied quantile regression, a statistical technique that allows investigation of possible relationships outside the mean response. We found several significant and mostly negative associations with CD, especially in lower quantiles of relative abundance on family or genus level. Associations found by quantile regression deviated from the mean response in relative abundances of Coriobacteriaceae, Pasteurellaceae, Peptostreptococcaceae, Prevotellaceae, and Ruminococcaceae. For the family Streptococcaceae we found a significant elevation in relative abundance for patients experiencing an exacerbation relative to those who remained without self-reported symptoms or measurable inflammation. Our analysis suggests that specific bacterial families are related to CD and exacerbation, but associations vary between patients due to heterogeneity in disease course, medication history, therapy response, gut microbiota composition and historical contingency. Our study underscores that microbial diversity is reduced in the gut of CD patients, but suggests that the process of diversity loss is rather irregular with respect to specific taxonomic groups. This novel insight may advance our ecological understanding of this complex disease.

## Introduction

Crohn’s disease (CD) is a chronic inflammatory disorder that can affect any part of the digestive tract, but mostly involves the ileum and colon.^1^ The disease is characterized by periods of inflammation (exacerbation) interspersed by periods without symptoms (remission). During exacerbation, the patients are suffering from a range of different symptoms, including diarrhea, abdominal pain, bloody stool, fatigue, and weight loss. Prolonged inflammation can lead to severe complications, like damage to the gastrointestinal tract and malnutrition.^1^ While the exact cause of CD is unknown, an inappropriate immune response against commensal gut bacteria, host genetics and environmental factors are all thought to be involved in disease pathophysiology.^2^ The gut microbiota in CD patients is characterized by a reduced diversity and lower long-term stability as compared to healthy individuals.^3^ Also, shifts in abundance of specific bacterial genera or families have been associated with CD^4^, its disease course^5^, and disease activity.^6^

Several studies have investigated relations between specific microbial groups and CD. *Faecalibacterium prausnitzii* (Ruminococcaceae), *Clostridium leptum* (Clostridiaceae), and *Clostridium coccoides* (Clostridiaceae) were found to be negatively associated with CD as well as disease activity.^4,6,7^ Conversely, the family Enterobacteriaceae was found to be positively associated with CD and with disease activity.^8,9^ However, the patterns of association with specific microbes are not always consistent among studies. Within the Bacteroidaceae family conflicting results were found. For example, CD patients showed both lower relative abundances^4,8^, as well as higher relative abundances^7^ in *Bacteroides* (Bacteroidaceae) compared to healthy individuals.

The inconsistency in findings might be partly due to technical artifacts, such as differences between studies in sequencing methods to quantify gut microbiota composition, and the compositional nature of data obtained by most next-generation sequencing (NGS) techniques. Another explanation is that the heterogenous responses among patients may derive from multi-factorial dependencies, between microbial elements themselves and between gut microbiota and host factors, such as treatment with immunomodulatory drugs, lifestyle, and diet, but also in underlying disease characteristics such as disease location, severity, and epigenetic immune regulation.^10,11^ This heterogeneity among patients is reflected by a strong variation in disease course, the response to medication, and the need for surgery among subgroups of patients.^12^ The involvement of specific bacterial groups in CD will likewise depend on multiple factors. Some of these factors can be accounted for when relating CD to gut microbiota composition, although correction relies on adequate model specification which is difficult in multi-factorial systems. Moreover, many factors which may strongly determine the observed relationships between bacterial abundance and CD activity have not been identified or are not routinely measured. One such factor is the order in which specific bacteria have been acquired throughout life. Rapid colonization by maternal and environmental bacteria occurs within days of birth and is unique per person. The temporal development of the microbiota is directed, implying that the growth of certain species precedes the growth of others, leading to the unique microbiomes in adult life. This historical contingency of gut microbiota might also influence how microbes react to future perturbations in that gut community.^13^

The multi-factorial involvement of specific microbial groups with CD necessitates robust analysis to uncover possible associations, as there may be no apparent or only weak relationships with the means of bacterial abundances. Here, we apply quantile regression, an extension of the general linear model that allows for investigation of relationships across different quantiles of the distribution of a response variable.^14,15^ Quantile regression extends regression of the mean to the analysis of the entire conditional distribution of the response variable.^15^ Examining quantile regression functions across the entire range of quantiles provides a more complete view of the response variable distribution than achieved by standard regression analysis.^14^ The idea behind this method is that not all individuals are equally responsive to changes in abundance of specific bacterial groups, due to hidden bias and complex dependencies in ecological datasets.^14^ Quantile regression is less sensitive to outliers than conventional regression and is not dependent on homoscedastic errors.^16^ In particular, we tested whether associations between relative abundances of specific families with CD can be found with a clinical diagnosis (i.e., remission vs. exacerbation), but also with specific markers (i.e., fecal calprotectin (FC), serum C-reactive protein (CRP), and the Harvey Bradshaw index (HBI)) of disease activity in repeatedly sampled CD patients and healthy controls.^6^

## Results

### Differences in abundance between healthy individuals and CD patients

We used the lqmm package (version 1.5.5)^17^ of the R statistical analysis software (http://www.R-project.org/) to perform the quantile regression analysis. Analyses were performed separately for each bacterial family. Although genus level might be preferred, this would have resulted in too many models. Therefore, we only looked at certain genus levels, when significant results were found at family level (within the base case selection as described in S2 Information). To accommodate repeated sampling on the individual level, we employed a linear quantile mixed model (LQMM) framework. A mixed model contains both fixed effects and random effects, and can then account for correlation in repeated measurements from the same individual as these are likely to be more similar than observations from different individuals.^18^ We estimated the series of quantile regression functions from the 10^th^ to the 90^th^ percent quantile. We used the Benjamini-Hochberg (BH) procedure per quantile to control for the expected proportion of “false discoveries” across microbial families.^19^ However, the BH procedure assumes independency in multiple testing, which is likely not the case in the gut microbiota. Therefore, the BH correction might provide too conservative estimates and we choose to also report the unadjusted results.

**Figure 1.**
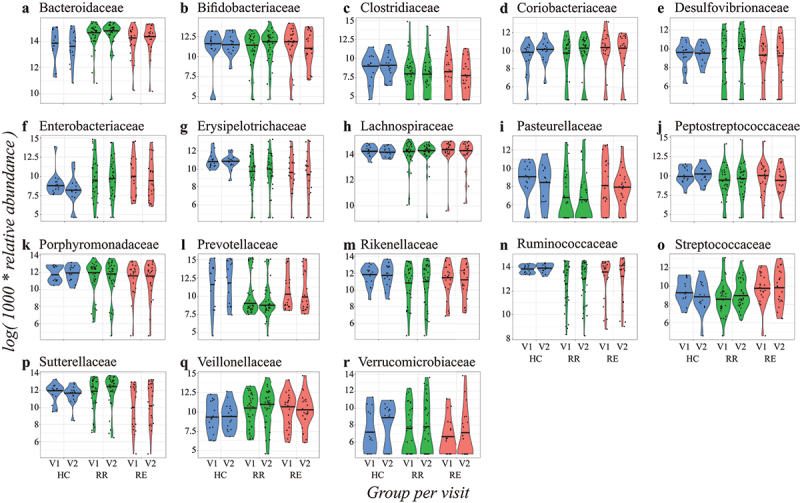
Violin plots of the transformed relative abundances of the base case bacterial families per group (genera in S4 Fig. and families outside the base case criterium in S7 Fig.). In blue the healthy controls, in green the RR group, and in red the RE group, all visualized per timepoint (V1 = visit 1 and V2 = visit 2). Patients in the RE group are in remission during the first visit and experience an exacerbation during the second visit. The 50% quantile is shown with a black horizontal line.

**Figure 2.**
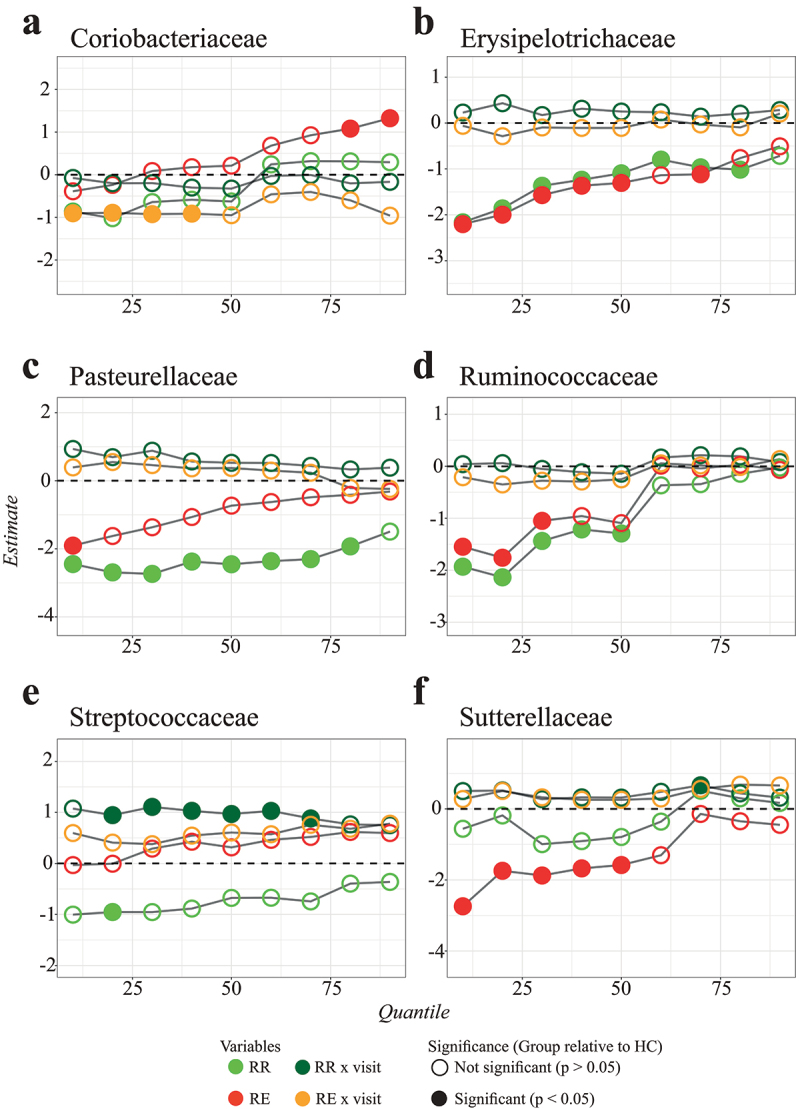
Examples of quantile regression profile plots for the families: Coriobacteriaceae (a), Erysipelotrichaceae (b), Pasteurellaceae (c), Ruminococcaceae (d), Streptococcaceae (e), and Sutterellaceae (f). The various dots represent sample estimates (y axis) of differences in relative abundance compared to healthy controls across the 10^th^ to the 90^th^ percent quantile (x axis). Differences at baseline (visit 1) are visualized in light green (RR) and red (RE), while the interaction with visit number (in dark green and orange) displays difference in changes over time. The dotted line at zero indicates no difference compared to healthy controls. When the points are above the dotted line there is a positive effect of disease group on relative abundance, whereas points below the dotted line imply a negative effect of disease group on relative abundance at that particular quantile. Significant variables (P-value <0.05) are indicated with a closed circle.

We found several associations between the relative abundances of bacterial families with CD, and more specific with remission or disease exacerbation (Figure 1). The quantiles that were significantly associated with CD are different per bacterial family. For example, patients with baseline sampling at time of remission and subsequent sampling during an exacerbation (RE) displayed significantly distinct distributions in relative abundance in the family Coriobacteriaceae (Figure 2a), both at baseline (visit 1) and at the second visit, compared to the healthy control subjects (HC). At baseline, there was a positive association in the higher quantiles and over time (at time of exacerbation) there was a negative association in the lower quantiles. This means that the distribution of Coriobacteriaceae abundance among RE patients is skewed to higher values at baseline, but to lower values at the follow-up visit, as compared to healthy controls (see also Figure 1d). However, these effects were no longer significant after Benjamini-Hochberg (BH) correction (S2 Fig.). For Coriobacteriaceae we also found a significant relation in the higher quantiles of patients in the RE group compared to the patients with two subsequent samples while maintaining remission (RR) (S3 Fig.). Thus, a significant fraction of patients in the RE group had higher Coriobacteriaceae abundance than healthy individuals and RR patients at baseline.

The family Erysipelotrichiaceae (Figure 2b) displayed negative associations in relative abundance over almost all quantiles (except the most upper quantiles) for both patient groups compared to the healthy controls. We still found significant differences after BH correction, but these were only present in the lowest quantiles (S2 Fig.). Looking at genus level, the genera *Holdemania* and *Turicibacter* displayed a similar pattern of significant results (S4 Fig., S5 Fig., and S6 Fig.). We did not find a significant difference among the patients in the RE and RR group (S3 Fig.). This implies that the relative abundance of Erysipelotrichiaceae is severely skewed to lower values in CD patients (see also Figure 1g). The same kind of relationship was also found for Ruminococcaceae (Figure 2d). Like Erysipelotrichiaceae, the associations were only found at baseline, suggesting that this is a characteristic of CD, but not related to disease activity.

As another example, the relative abundances of Sutterellaceae among patients in the RE group were significantly skewed to lower values compared to both the healthy controls and the RR group patients (Figures 2f, 1p, and S3 Fig.). We also found a significant negative relation between the abundance of the family Pasteurellaceae in the RR group at baseline compared to the healthy controls (Figure 2c). For the family Streptococcaceae, we did not find many significant associations at baseline (except for one quantile), but the association for the RR x visit variable was significant for almost all quantiles (Figure 2e). This means that patients from the RR group experienced stronger increases in relative abundance of Streptococcaceae over time as compared to the healthy controls. We also found a significant difference between the RR and RE patient groups for the family Streptococcaceae, with the RE patients having elevated abundances across the entire quantile range (S3 Fig., see also Figure 1o). In our data, the Pasteurellaceae and Streptococcaceae both only consisted of one classified genus. When refining the analyses for these classified genera, we did not find significant results within *Mannheimia* (Pasteurellaceae) or *Streptococcus* (Streptococcaceae) (S4 Fig., S5 Fig., and S6 Fig.).

**Figure 3.**
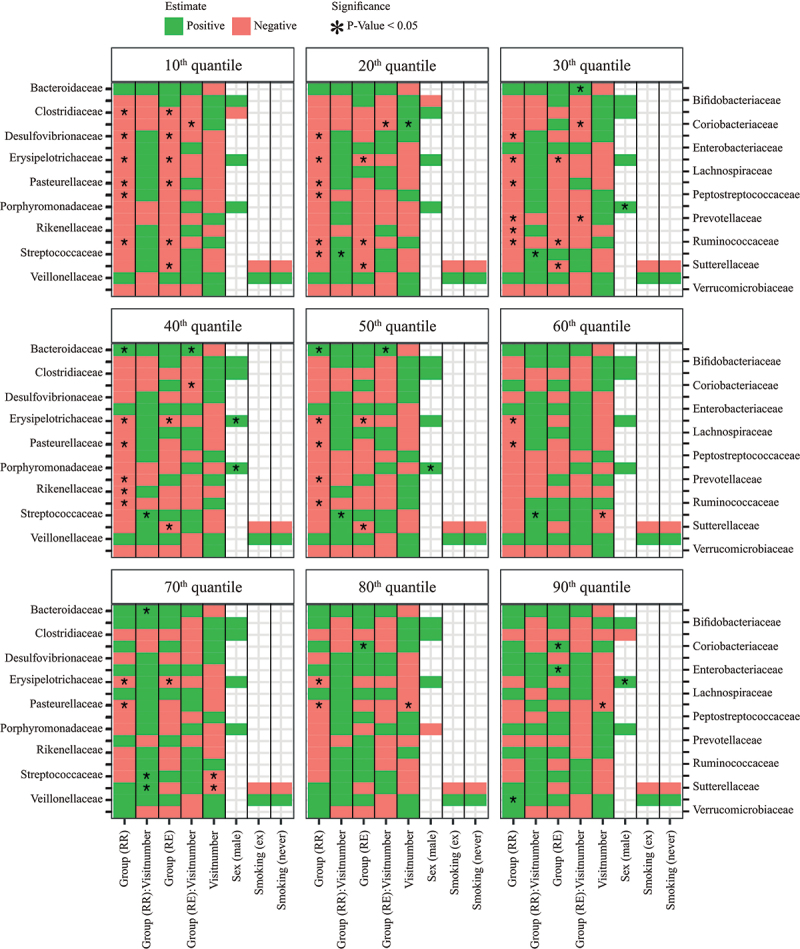
Heatmap of quantile regression estimates per quantile of relative abundance for base case families (other families outside the base case criterium in S8 Fig. and S9 Fig.) and common variables. The model included all groups, i.e. healthy control (HC), remission-remission (RR), and remission-exacerbation (RE), with healthy controls as reference group. The red boxes indicate negative regression estimates, the green boxes indicate positive regression estimates, and the empty boxes are the variables that were not selected during variable selection. Significant variables (P-value <0.05) are indicated with an asterisk (“*”), results adjusted with the BH procedure are given in S2 Fig.

On top of the examples given above, we also found that the relative abundances of Clostridiaceae, Desulfovibrionaceae, Peptostreptococcaceae, Prevotellaceae, and Rikenellaceae in CD patients were different from the relative abundances in the microbiota of healthy controls (Figure 3). These results, except for the families Prevotellaceae and Rikenellaceae, remained significant after BH correction (S2 Fig.). Most significant relations were negative and were found in the lower quantiles (Figure 3), meaning that CD patients more often displayed negatively than positively skewed abundance distributions (see also Figure 1). Besides, only a few associations between bacterial family abundance and covariates were found, with sex being the only significant covariate (males having higher abundances than females) (Figure 3). We also identified some significant associations in the families which fall under the sensitivity analyses of the families outside the base case selection criterium (S7 Fig., S8 Fig., and S9 Fig.). We found that the relative abundances of Victivallaceae and Clostridiales I.S. XI were different from the healthy controls for both the patients that stayed in remission and the patients that experienced an exacerbation. We also found significant results for the family Enterococcaceae for the patients that stayed in remission compared to the healthy controls and the families Actinomycetaceae and Lactobacillaceae for the patients that experienced an exacerbation at the second visit (S8 Fig.). However, these results disappeared after applying BH correction for multiple testing (S9 Fig.).

We compared our results from the LQMM models with the results obtained from an ordinary linear mixed effect model (with similar variables as used in the LQMM models) by using the lme function from the nlme package (version 3.1)^20^ in R. Example code of the LQMM and LME models can be found on the Github repository (https://github.com/susannepinto/Quantile-Regression-CD). Most associations found by quantile regression could also be found with ordinary regression, as the mean response in the linear mixed effect model provides somewhat of an average response over all quantiles. Nevertheless, some differences were also noticeable (S10 Fig.). For example, the family Coriobacteriaceae has a positive estimate in the higher quantiles for patients in the RE group relative to the healthy control group, which is not visible in the mean response (S10D Fig.). Likewise, patients in the RR group displayed significant reductions in abundance in the lower to middle quantiles of Prevotellaceae and Streptococcaceae abundance, that were apparent in a reduced mean response, but without statistical significance. Conversely, the reduced mean responses regarding Ruminococcaceae in both RR and RE patients hide the fact that reductions only apply to lower and middle quantiles of abundance (Figure 1n and S10N Fig.). Comparable findings were obtained for Pasteurellaceae and Peptostreptococcaceae. In some instances, linear mixed effect regression yielded imprecise (cf. Lachnospiraceae) or biased (cf. group x visit in Prevotellaceae) estimates as compared to quantile regression (S10 Fig.).

### Gut microbiota changes in relation to Crohn’s disease activity

**Figure 4.**
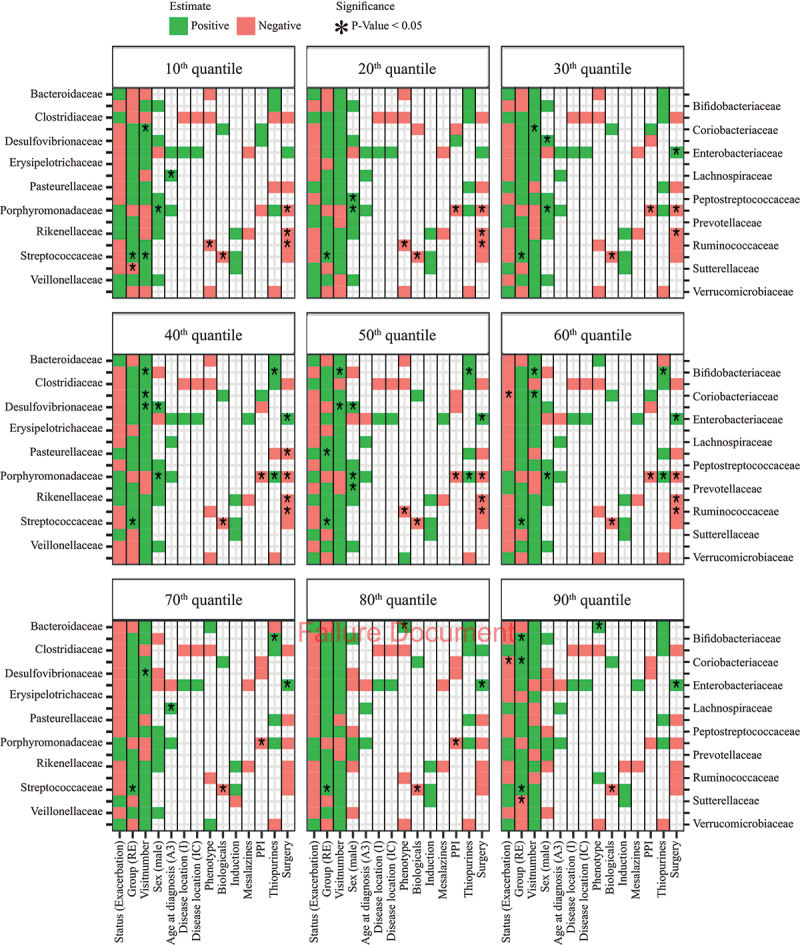
Heatmap of quantile regression estimates per quantile of relative abundance for base case families for CD patients only, with correction for clinical variables. The red boxes indicate negative regression estimates, the green boxes indicate positive regression estimates, and the empty boxes are the variables that were not selected during variable selection. Significant variables (P-value <0.05) are indicated with an asterisk (“*”), results adjusted with the BH procedure are given in S11 Fig.

The relation between bacterial family abundance and disease activity (exacerbation) was mainly negative across the quantile range, indicating reduced abundance among RE patients as compared to RR patients at the 2^nd^ visit. However, this association was only statistically significant for upper quantiles of Coriobacteriaceae after adjustment for covariates (Figure 4). Instead, significant associations were revealed with several clinical variables (e.g., phenotype, surgery, proton pump inhibitors (PPI), and biologicals), suggesting that differences between RR and RE patients might have been confounded by disease-specific variables (Figure 4). Of note, many associations with disease activity also disappeared after BH correction for multiple testing (S11 Fig.), but the finding that RE patients had elevated Streptococcaceae abundances across the entire quantile range (irrespective of visit number) remained significant, just as treatment with biologicals remained significantly associated with lower Streptococcaceae abundance (Figure 4 and S11 Fig.). Further results on genera and other families can be found in S12 to S15 Fig.

### Bacterial relative abundances in relation to different disease activity indicators

**Figure 5.**
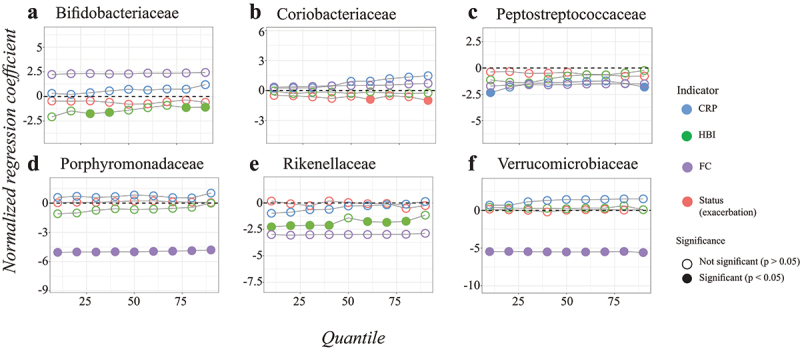
Quantile regression profile plots obtained for the different disease activity indicators with correction for clinical variables for the families: Bifidobacteriaceae (a), Coriobacteriaceae (b), Peptostreptococcaceae (c), Porphyromonadaceae (d), Rikenellaceae (e), and Verrucomicrobiaceae (f). Only families with significant findings are given, the other families are in S16 Fig. The dotted line at zero indicates no difference compared to healthy controls. When the points are above the dotted line there is a positive effect of disease group on relative abundance, whereas points below the dotted line imply a negative effect of disease group on relative abundance at that particular quantile. The regression estimates for status, HBI, CRP, and FC were estimated in different models, therefore the data was normalized beforehand to make the regression coefficients comparable. On this purpose, the values for HBI, CRP, and FC were divided by the difference between the 5^th^ and 95^th^ percentiles. Significant variables (P-value <0.05) are indicated with a closed circle, results adjusted with the BH procedure are given in S17 Fig.

When comparing regression onto clinically defined exacerbation with different indicators of disease activity, we found that especially FC levels gave distinct results compared to the other indicators (Figure 5, S16 Fig.). For almost all bacterial families, we did not observe a signal (estimates around zero) for clinical status (remission or exacerbation), CRP, and HBI after correction for clinical variables. In contrast, the normalized estimates of FC were much stronger (S16 Fig.), and significantly negative across the entire quantile range for Porpyromonadaceae and Verrucomicrobiaceae (Figure 5). The results of Porphyromonadaceae still hold after BH correction (S17D Fig.). Further results on genera and other families can be found in S18 to S21 Fig.

## Discussion

In this study, we investigated the possible associations of the relative abundance of specific bacterial families with CD and disease activity. We relied on quantile regression to uncover relationships that are not restricted to the mean response across CD patients. We found mainly negative associations with CD at the family or genus level, especially in lower quantiles of relative bacterial abundance. These results are consistent with the frequently cited reduced microbial diversity in the gut of CD patients compared to healthy controls, but they also highlight that reductions for specific microbes are usually limited to a minority of patients. Thus, while CD coincides with a loss of microbial diversity, the process of diversity loss seems rather irregular with respect to specific taxonomic groups.

Associations outside of the mean response can be a result of heterogeneity among CD patients and may arise from the complex interactions among members of the microbiota. Microbes alter the environment through metabolic by-products, creating new ecological niches that promote diversification. However, some metabolites may adversely affect the growth of other microorganisms.^21^ Whether systemic changes, such as those induced by CD, lead to niche reduction or expansion for a particular microbe probably depends as much on the characteristics of that particular microbe as on the microbial ecosystem of the individual host. If specific bacterial groups respond to disease or disease activity in some of the patients but not in others, their associations with CD or disease activity are more likely to be found in upper or lower quantiles than in the mean or median response across CD patients.

Interestingly, almost all the significant associations we found were negative and applicable to the lower quantiles of bacterial abundance. While positive associations in upper quantiles have been attributed to unmeasured factors that limit the potential response to a positive stimulus,^14^ this opposite pattern is reminiscent of ecosystem response to stress: the ability to maintain healthy bacterial abundances is gradually lost once the system gets close to a tipping point.^22^ The loss of some species in the microbial network can still be compensated for by other species with similar ecosystem functions (functional redundancy), but the loss of too many may lead to a loss of resilience and critical transition to an alternative stable state.^23,24^ Although the existence of tipping points in the onset or exacerbation of CD has not been demonstrated, a large-scale study by Lahti et al. (2014) showed distinct bimodal abundance patterns of certain bacterial species (i.e., tipping elements) among healthy human hosts. These species were present in either a high or low abundance state, supporting the idea of alternative stable states in the human gut microbiota.^25^ Taken together, concepts of ecosystem resilience and critical transitions in the gut microbiota may explain why some individuals respond strongly to systemic changes, as induced by CD, while others display a more robust microbiota composition.^11^

Associations between the relative abundance of bacterial families and CD across the entire quantile range can also be identified with methods that focus on the mean response, such as ordinary linear regression. Families that exhibit such uniform responses could be considered to represent keystone bacterial groups, as their response to CD or disease activity is less dependent on other microbes or host factors as compared to families that are only responsive in some patients. This feature of robustness would be preferred for clinical diagnostics, prioritization for treatment or monitoring of disease course, because guidelines can then be developed and used for all CD patients. However, it is also important to understand the less generic differences in the microbiota of CD patients because less robust associations may also shed light on etiology and progression of CD and may provide leads for personalized treatment strategies. This is especially important considering the heterogeneous disease course, therapy response, and potentially contributing factors to microbiota perturbations. Moreover, the results of our analysis might help to reconcile inconsistencies in previously published findings as regards involvement of specific bacterial families in CD.

Our results confirm previously identified associations of CD with the families Erysipelotrichaceae, Peptostreptococcaceae, Prevotellaceae, Rikenellaceae, Ruminococcaceae (e.g. *Faecalibacterium prausnitzii*), and Veillonellaceae.^6,8,9^ In addition, we also identified previously unreported associations between CD and the families Coriobacteriaceae, Desulfovibrionaceae, Streptococcaceae, and Sutterellaceae. Mixed results (both negative and positive associations) have been reported for the family Bacteroidaceae (e.g. *Bacteroides fragilis*).^7–9^ Our results suggest that these mixed results can be explained by a change in association (from positive to negative) across the quantile range. Likewise, we found a negative association with the family Pasteurellaceae when the patients were compared to healthy individuals, especially with regards to patients who remained in remission. This is in contrast with previous results showing a positive association between relative abundances of Pasteurellaceae and CD.^9^

Previous studies did not make use of quantile regression to identify possible associations between the microbiota and factors related to inflammation. Most studies compared samples based on their means or medians (by student’s t-test, Mann-Whitney U test or the analysis of variance), without taking into account the confounding effect of covariates, such as medication use or the age of the patient.^4,7,8,26^ Other studies used methods that can take covariates into account, such as generalized linear regression models, but these still only consider the mean count or relative abundance and do not consider distinct associations across patients.^7,9,26,27^ Lastly, supervised classifiers (e.g. Random Forest) and clustering algorithms (e.g. agglomerative hierarchical clustering) are used to predict the presence or activity of disease by the pattern in relative abundance of many families at once.^6^ While these methods are not constrained by the strict assumptions of regression models, they have difficulties in dealing with repeated measurements and covariates. In addition, these methods require many patients, which are often not available in longitudinal clinical cohorts.

A practical advantage of quantile regression is its usefulness in situations when assumptions of other methods are violated. For example, quantile regression does not require homoscedastic and normally distributed data. On the contrary, the method enables to detect and describe changes in the conditional distribution of the response variable when there is heteroscedasticity, skewness, or kurtosis in the data.^14^ Another limitation of quantile regression is that it is hard to use for the purpose of prediction. Nevertheless, quantile regression is powerful when heterogeneous response distributions should be expected, e.g. if many interdependencies and potentially limiting factors play a role. If those co-factors are differently distributed among patients and not included in the model, they lead to (hidden) bias in conventional regression but can be dealt with in quantile regression.^28^

We found several significant associations of bacterial abundance with the presence of CD. However, associations with disease activity were less evident in our data. Although we found some differences among the two groups of CD patients, we only found significantly elevated abundances for Streptococcaceae (at baseline) and Coriobacteriaceae (during active disease) in patients experiencing an exacerbation relative to patients remaining in remission, and the latter effect disappeared after correction for multiple testing. Multiple explanations are possible for the lack of significant associations between exacerbation and remission. Firstly, the microbiota of CD patients might not be responsive to exacerbation as compared to remission. Multiple studies underline our finding of no clear significant differences between remission and active disease.^5,6^ In other words, the observed differences in bacterial abundances are disease-related community differences that even persist in the absence of active inflammation, and therefore this pattern is not significantly reflected between the disease states.^29,30^ However, most studies, including ours, also likely lacked the statistical power to find such potential differences. A second possibility is that potential associations are confounded with other factors that are likely to play a role in shaping the microbiota, such as disease severity^27^, disease duration^5^, disease location^26^, treatments^31^, and host characteristics (such as smoking).^32^ Treatments such as PPIs have been shown to change gut microbiota and individuals undergoing surgery will have been given antibiotics to prevent infection.^31^ Lastly, we might not have looked at the right taxonomic level, while the resolution of taxonomic profiling could impact the accuracy and specificity of our findings. Most differences are possibly only present at species or strain levels, or even require metabolic/functional analysis.^11,32,33^ Moreover, a change in relative abundance at taxonomic level might not reflect a change in ecosystem functioning, as expansion in certain species can compensate for the loss of another (functionally similar) species.^11^ Nonetheless, we did find stronger associations with fecal calprotectin than with the clinical definition of CD activity. This suggest that a quantitative measure of inflammation carries information about the microbial involvement in disease activity. As the level of fecal calprotectin is only a proxy of inflammation in the gut, the associations might become even more clear when specific immunological markers, or even hormones, would be used.

It is important to acknowledge several limitations that may impact the generalizability and interpretation of our findings. Firstly, our study was conducted within a relatively small cohort of CD patients (*n* = 57) and healthy controls (*n* = 15). While this cohort size allowed us to perform longitudinal analyses, it may limit the generalizability of our results to broader CD populations. Also, quantile regression is not insensitive to outliers, especially in the highest and lowest quantiles when there is not much data left for estimation, potentially affecting the robustness of our statistical analyses. With a larger dataset, one might consider a finer quantile division to obtain a smoother quantile regression profile, while with a smaller dataset, a coarser division would be more appropriate. Furthermore, as with any observational study, causation cannot be inferred from our results, and further mechanistic investigations are needed to elucidate whether there is a role of the highlighted bacterial families in CD pathogenesis. Lastly, the dynamic nature of the gut microbiota and potential temporal variations were not extensively explored in this study, which might have limited our ability to capture the full spectrum of microbial changes associated with CD over time. In light of these limitations, caution should be exercised when interpreting our results, and future research hopefully addresses these constraints through larger, more diverse cohorts and a finer taxonomic resolution to provide a more comprehensive understanding of the gut microbiota’s role in CD.

In this study we showed that associations of CD with relative bacterial abundances can be different for subsets of individuals. Our findings revealed significant negative associations with CD for several bacterial families such as Pasteurellaceae, Peptostreptococcaceae, Prevotellaceae, and Ruminococcaceae, highlighting their potential roles in CD pathogenesis. Furthermore, the significant differences in the relative abundance of Sutterellaceae and Streptococcaceae among CD patients who experienced exacerbations, relative to those who maintained remission, were not seen before and underscore the dynamic nature of microbial associations in relation to disease activity. The subtle variations observed in the family Coriobacteriaceae, which could not be seen in the mean response, further emphasize the complexity of these relationships. Importantly, our study underscores the heterogeneity of CD and its impact on gut microbiota, suggesting that associations may only become evident when considering patients’ diverse disease courses, medication histories, therapy responses, and gut microbiota compositions. Associations with specific bacterial families may only be detectable in a minority of patients, hence they cannot generally be considered to identify CD or disease activity. The novelty of our study lies in its rigorous approach to exploring associations in subsets of patients, acknowledging the heterogeneity between them. In such situations, quantile regression is a useful tool for distilling potential relationships, that may remain unidentified by commonly used methods. We recommend its use in even larger cohorts, to get a better understanding of CD in relation to the gut microbiota.

## Methods

### Data and procedures

The study population has previously been described in Galazzo et al. (2019).^6^ A total of 57 CD patients were included in this study. Demographic variables and subject characteristics are provided in S1 Table and medication use between visits is provided in S2 Table. The CD patients formed a subset of the Inflammatory Bowel Disease South Limburg Cohort.^34^ As a reference group, 15 HC subjects, all without any gastrointestinal disease, gastrointestinal symptoms, or comorbidities, were recruited among the controls who participated in the Maastricht Irritable Bowel Syndrome (IBS) Cohort.^35^ Clinical data, blood, and feces were collected at two timepoints. The CD group comprised 22 RE patients with baseline sampling at time of remission and subsequent sampling during an exacerbation, and 35 RR patients, with two subsequent samples while maintaining remission, i.e. without any flares in between subsequent samples. The median time between baseline and follow-up samples was 14 [IQR 11–21], 20 [8–36], and 13 [12–16] weeks for RR patients, RE patients, and HCs, respectively (S2 Table). All study subjects gave written informed consent prior to participation. Both studies have been approved by the Medical Ethics Committee of Maastricht University Medical Center and have been registered in the US National Library of Medicine [http://www.clinicaltrials.gov: NCT02130349 and NCT00775060, respectively].

Fecal samples were collected at home, kept at room temperature, and brought to the hospital within 12 hours after defecation. Part of the fecal sample of the CD patients was sent to the laboratory of Clinical Chemistry for routine analysis of FC. The remaining part was aliquoted and frozen at −80°C for microbiota analysis. Disease activity was defined by FC, serum CRP, and HBI. Patients were included in the study when patients were in remission at baseline, i.e. FC <100 μg/g and CRP <5 mg/L or FC <100 μg/g, CRP <10 mg/L, and HBI ≤ 4. Exacerbation at the second timepoint was defined by FC >250 μg/g or FC >100 μg/g with at least a 5-fold increase from baseline (S1 Fig.). The fecal microbiota composition was assessed by Illumina Miseq sequencing of the V4-region of the 16S rRNA gene. A detailed description of metagenomic DNA isolation, sequencing, and quality control is provided in the supplementary information of Galazzo et al. 2019.^6^ The 16S rRNA gene sequencing data are released in the European Nucleotide Archive. The accession number is: PRJEB62578 ERP147674.

Information on microbial profiling and the selection of bacterial families for quantile regression analysis can be found in the appendix (S1 information and S2 information).

### Linear Quantile Regression Mixed Models (LQMM)

The quantile regression model takes the form $Q_{Y|X}\left(\tau \right)=X\beta_{\tau }$, where $Q_{Y|X}\left(\tau \right)$ denotes the τ^th^ quantile of the response variable Y, which is predicted from a vector X of explanatory variables with quantile-specific parameters $\beta_{\tau }$. The τ^th^ quantile is the inverse of the cumulative distribution function of Y, i.e. $q_{Y}\left(\tau \right)=F_{y}^{-1}\left(\tau \right)$ or reciprocally $F_{Y}\left(q_{\tau }\right)=P\left(Y\leq y\right)=\tau ,where\;\tau \in \left[0,1\right]$. It denotes the smallest value where the probability of finding an even smaller value is less than or equal to τ, whereas the probability of finding a larger value is less than or equal to 1 − τ .^14^ A parametric distribution is assumed for the deterministic part of the model, but the random error part does not assume any distributional form. Further information on inclusion of covariates and model building strategy is supplied in the appendix (S3 information).

## Supplementary Material

Supplemental Material

## Data sharing statement

The study protocol of the South Limburg IBD cohort in which the current study was embedded has been published previously: https://academic.oup.com/ije/article/46/2/e7/3038105. All study subjects gave written informed consent prior to participation. Both studies have been approved by the Medical Ethics Committee of Maastricht University Medical Center and have been registered in the US National Library of Medicine (http://www.clinicaltrials.gov): NCT02130349 and NCT00775060, respectively. The 16S rRNA gene sequencing data are released in the European Nucleotide Archive. The accession number is: PRJEB62578 ERP147674. Example code of the LQMM and LME models can be found on the Github repository: https://github.com/susannepinto/Quantile-Regression-CD.
