# Supplementary material for: Heterogeneous associations of gut microbiota with Crohn’s disease activity
Source: Gut Microbes. 2023 Dec 17;16(1):2292239. doi: 10.1080/19490976.2023.2292239 (PMC10730216; doi:10.1080/19490976.2023.2292239)
Supplement: Supplemental Material [file KGMI_A_2292239_SM2492.docx]

**Supplementary materials**

Table of Contents

[Data description 3](#_Toc145141184)

[S1 Table. Descriptive statistics 3](#_Toc145141185)

[S2 Table. Medication use and time between sampling moments for remission and exacerbation samples 5](#_Toc145141186)

[Extra information on data and procedures 6](#_Toc145141187)

[S1 Information. Data procedures 6](#_Toc145141188)

[S2 Information. Family selection 6](#_Toc145141189)

[S3 Information. Model building strategy 6](#_Toc145141190)

[Disease indicators 8](#_Toc145141191)

[S1 Figure. Disease indicators 8](#_Toc145141192)

[Sensitivity analyses: Differences in abundance between healthy individuals and CD patients 9](#_Toc145141193)

[S2 Figure. Results obtained with quantile regression for base case families and variables, adjusted with the Benjamini-Hochberg procedure. 9](#_Toc145141194)

[S3 Figure. Results obtained with quantile regression for base case families and variables 10](#_Toc145141195)

[S4 Figure. Violin plots of the transformed relative abundances of the selected genera per group. 11](#_Toc145141196)

[S5 Figure. Results obtained with quantile regression for the selected genera and variables. 12](#_Toc145141197)

[S6 Figure. Results obtained with quantile regression for the selected genera and variables, adjusted with the BH procedure. 13](#_Toc145141198)

[S7 Figure. Violin plots of the transformed relative abundances of the families outside the base case selection criterium per group. 14](#_Toc145141199)

[S8 Figure. Results obtained with quantile regression of the families outside the base case selection criterium and variables. 15](#_Toc145141200)

[S9 Figure. Results obtained with quantile regression of the families outside the base case selection criterium and variables, adjusted with the BH procedure. 16](#_Toc145141201)

[S10 Figure. Comparing the results of the LQMM analysis (20%, 50%, 80% quantiles) with the results of the linear mixed effect models. 17](#_Toc145141202)

[Sensitivity analyses: Gut microbiota changes in relation to Crohn’s disease activity 18](#_Toc145141203)

[S11 Figure. Results obtained with quantile regression for base case families for CD patients cohort data only, with correction for clinical variables and p-value adjustment according BH procedure. 18](#_Toc145141204)

[S12 Figure. Results obtained with quantile regression for the selected genera and variables for CD patients cohort data only, with correction for clinical variables. 19](#_Toc145141205)

[S13 Figure. Results obtained with quantile regression for the selected genera and variables for CD patients cohort data only, with correction for clinical variables and p-value adjustment according BH procedure. 20](#_Toc145141206)

[S14 Figure. Results obtained with quantile regression of the families outside the base case selection criterium for CD patients cohort data only, with correction for clinical variables. 21](#_Toc145141207)

[S15 Figure. Results obtained with quantile regression of the families outside the base case selection criterium for CD patients cohort data only, with correction for clinical variables and correction with the BH procedure. 22](#_Toc145141208)

[Sensitivity analyses: Bacterial families relative abundances in relation to different disease activity indicators 23](#_Toc145141209)

[S16 Figure. Results obtained for the different disease activity indicators with correction for clinical variables. 23](#_Toc145141210)

[S17 Figure. Results obtained for the different disease activity indicators with correction for clinical variables, with p-value adjustment according BH procedure. 24](#_Toc145141211)

[S18 Figure. Results obtained for the different disease activity indicators for the selected genera and variables for CD patients cohort data only. 25](#_Toc145141212)

[S19 Figure. Results obtained for the different disease activity indicators for the selected genera and variables and p-value adjustment according BH procedure for CD patients cohort data only. 26](#_Toc145141213)

[S20 Figure. Results obtained for the different disease activity indicators of the families outside the base case selection criterium and variables for CD patients cohort data only. 27](#_Toc145141214)

[S21 Figure. Results obtained for the different disease activity indicators of the families outside the base case selection criterium and variables and p-value adjustment according BH procedure for CD patients cohort data only. 28](#_Toc145141215)

# Data description

|  | HC (N=30) | RR (N=70) | RE (N=44) | Overall (N=144) |
| --- | --- | --- | --- | --- |
| **Sex** |  |  |  |  |
| female | 14 (46.7%) | 50 (71.4%) | 24 (54.5%) | 88 (61.1%) |
| male | 16 (53.3%) | 20 (28.6%) | 20 (45.5%) | 56 (38.9%) |
| **Smoking** |  |  |  |  |
| ex | 4 (13.3%) | 34 (48.6%) | 20 (45.5%) | 58 (40.3%) |
| never | 26 (86.7%) | 20 (28.6%) | 20 (45.5%) | 66 (45.8%) |
| current | 0 (0%) | 16 (22.9%) | 4 (9.1%) | 20 (13.9%) |
| **Age** |  |  |  |  |
| Mean (SD) | 26.7 (5.93) | 42.6 (12.8) | 43.6 (16.8) | 39.6 (14.7) |
| Median [Min, Max] | 25.0 [20.0, 45.0] | 43.0 [17.0, 67.0] | 42.5 [19.0, 68.0] | 38.5 [17.0, 68.0] |
| **Disease location^1^** |  |  |  |  |
| C | NA | 16 (22.9%) | 14 (31.8%) | 30 (20.8%) |
| I | NA | 24 (34.3%) | 14 (31.8%) | 38 (26.4%) |
| CI | NA | 30 (42.9%) | 16 (36.4%) | 46 (31.9%) |
| **Age at diagnosis^2^** |  |  |  |  |
| A2 | NA | 62 (88.6%) | 28 (63.6%) | 90 (62.5%) |
| A3 | NA | 8 (11.4%) | 16 (36.4%) | 24 (16.7%) |
| **Surgery** |  |  |  |  |
| 0 | 30 (100%) | 54 (77.1%) | 36 (81.8%) | 120 (83.3%) |
| 1 | 0 (0%) | 16 (22.9%) | 8 (18.2%) | 24 (16.7%) |
| **Phenotype** |  |  |  |  |
| 0 | NA | 52 (74.3%) | 24 (54.5%) | 76 (52.8%) |
| 1 | NA | 18 (25.7%) | 20 (45.5%) | 38 (26.4%) |
| **Mesalazines** |  |  |  |  |
| 0 | 30 (100%) | 60 (85.7%) | 35 (79.5%) | 125 (86.8%) |
| 1 | 0 (0%) | 10 (14.3%) | 9 (20.5%) | 19 (13.2%) |
| **Thiopurines** |  |  |  |  |
| 0 | 30 (100%) | ^[[1]](#footnote-1)^46 (65.7%) | 28 (63.6%) | 104 (72.2%) |
| 1 | 0 (0%) | 24 (34.3%) | 16 (36.4%) | 40 (27.8%) |

S1 Table. Descriptive statistics (N_individuals_ = 72). Note that two samples per individual were collected

| **Biologicals^3^** |  |  |  |  |
| --- | --- | --- | --- | --- |
| 0 | 30 (100%) | 32 (45.7%) | 15 (34.1%) | 77 (53.5%) |
| 1 | 0 (0%) | 38 (54.3%) | 29 (65.9%) | 67 (46.5%) |
| **Induction** |  |  |  |  |
| 0 | 30 (100%) | 57 (81.4%) | 36 (81.8%) | 123 (85.4%) |
| 1 | 0 (0%) | 13 (18.6%) | 8 (18.2%) | 21 (14.6%) |
| **PPI^4^** |  |  |  |  |
| 0 | 30 (100%) | 56 (80.0%) | 28 (63.6%) | 114 (79.2%) |
| 1 | 0 (0%) | 14 (20.0%) | 16 (36.4%) | 30 (20.8%) |
| **HBI^5^** |  |  |  |  |
| Mean (SD) | NA | 2.41 (2.95) | 3.00 (3.39) | 2.08 (2.97) |
| Median [Min, Max] | NA | 1.00 [0, 11.0] | 2.00 [0, 13.0] | 1.00 [0, 13.0] |
| Missing | NA | 0 (0%) | 2 (4.5%) | 2 (1.4%) |
| **CRP^6^** |  |  |  |  |
| Mean (SD) | NA | 2.57 (2.05) | 3.76 (3.28) | 2.31 (2.61) |
| Median [Min, Max] | NA | 2.00 [0.900, 11.0] | 2.80 [0.900, 13.0] | 1.40 [0, 13.0] |
| Missing | NA | 5 (7.1%) | 8 (18.2%) | 13 (9.0%) |
| **FC^7^** |  |  |  |  |
| Mean (SD) | NA | 28.6 (20.9) | 290 (742) | 102 (426) |
| Median [Min, Max] | NA | 14.0 [14.0, 98.0] | 110 [14.0, 4900] | 15.0 [0, 4900] |

**^[[2]](#footnote-2)^**

|  | RR |  | RE |  |
| --- | --- | --- | --- | --- |
|  | Remission  (n = 35) | Remission  (n = 35) | Remission  (n = 22) | Exacerbation  (n = 22) |
| **Medication^1^** |  |  |  |  |
| Mesalazine | 5 (14.3%) | 5 (14.3%) | 4 (18.2%) | 5 (22.7%) |
| Thiopurines | 11 (31.4%) | 11 (31.4%) | 9 (40.9%) | 7 (31.8%) |
| Biologicals | 18 (51.4%) | 19 (54.3%) | 13 (59.1%) | 15 (68.2%) |
| Corticosteroids | 1 (2.9%) | 0 (0%) | 1 (4.5%) | 1 (4.5%) |
| PPI | 7 (20%) | 7 (20%) | 8 (36.4%) | 8 (36.4%) |
| Antibiotics^2^ | 1 (2.9%) | 0 (0%) | 1 (4.5%) | 0 (0%) |
| Time between sampling moments (week, median, IQR) | 14 [11-21] | | 20 [11-21] | |
|  |  | ^[[3]](#footnote-3)^4 |  |  |

## S2 Table. Medication use and time between sampling moments for remission and exacerbation samples

# Extra information on data and procedures

## S1 Information. Data procedures

Data demultiplexing, length and quality filtering, and clustering of reads into Operational Taxonomic Units (OTUs) at 97% sequence identity was done using the online Integrated Microbial Next Generation Sequencing (IMNGS) platform using default settings except for minimum and maximum length for amplicons, which were set at 100 and 500 bp respectively.^1^ After quality filtering, binning, and removing unassigned reads, sequences were clustered in 640 OTUs. Normalization was performed by dividing OTU counts per sample for their total count (sample depth) and then multiplying the obtained relative abundance for the lowest sample depth.

## S2 Information. Family selection

First, OTU sequences assigned to chloroplasts were removed prior to the statistical analyses. Then, the 18 different families used in the main text were selected by removing rare reads (not seen more than three times in at least 20% of the samples). We also performed quantile regression on the remaining families (which were not selected by the base case threshold), these results are placed in the supplementary materials.

## S3 Information. Model building strategy

The analysis was divided in three parts. First, we investigated whether the relative abundances of the bacterial families could be explained by the group to which each individual belongs (i.e., healthy control (HC), remission-remission (RR), or remission-exacerbation (RE)). We added the interaction with visit number, to allow for different temporal changes in bacterial relative abundance over time between healthy controls, CD patients who experienced an exacerbation at the second visit, and those who remained in remission. Secondly, we excluded the healthy individuals from the model and investigated whether the relative abundances of the bacteria could be explained by disease activity (i.e., remission vs. exacerbation) in the CD patient group. Thirdly, we additionally investigated if the relative abundances of the bacterial families could be related to a quantitative disease indicator (i.e., HBI, CRP, or FC) other than the clinical definition of disease activity (remission and exacerbation). The models contain two timepoints per individual. Therefore, we used a random intercept per patient as well as a random effect for the variable ‘visit number’, because temporal changes in bacterial family’s relative abundance may differ within patients, even when accounting for the fixed effect of disease trajectory (e.g., experiencing an exacerbation at the second visit).

Prior to the analyses, relative abundances were multiplied with 1000 and log-transformed with the natural log function assuming a lower detection limit of 100 reads (which is 1/4^th^ of the lowest measurable value in the data). Prior to variable selection, all models contained the variables sex (male vs. female), smoking (current, ex, or never), and age (centered around mean age of 39,6 years) (S1 Table). The models for CD patient cohort data also contained the disease-specific variables disease location (colonic (C), ileal (I), or ileocolonic (IC)), age at diagnosis (younger than 40 years (0) or older than 40 years (1)), surgery (no (0) vs. yes (1)), disease phenotype (non-stricturing/non-penetrating vs. stricturing/penetrating ), and current treatment (mesalazines (no (0) vs. yes (1)), thiopurines (no (0) vs. yes (1)), biologicals (no (0) vs. yes (1)), induction (no (0) vs. yes (1)), and proton pump inhibitors (PPI) (no (0) vs. yes (1))) (S1 Table). Variable selection was performed by running all possible models and then selecting the model with the lowest Bayesian Information Criterion (BIC) in the 50% quantile. For the model with the disease indicators (HBI, CRP, and FC), variable selection was performed on a model that contained all three indicators. For the sake of comparison, the selected variables were also used in the separate models for HBI, CRP, and FC. FC was divided by 1000 to improve numerical precision in quantile regression. Moreover, HBI, CRP, and FC are measured on different scales, therefore the data was normalized beforehand to make the quantile regression model estimates comparable. On this purpose, the values for HBI, CRP, and FC were divided by the difference between the 5^th^ and 95^th^ percentiles.

References

1. Lagkouvardos I, Joseph D, Kapfhammer M, Giritli S, Horn M, Haller D, et al. IMNGS: A comprehensive open resource of processed 16S rRNA microbial profiles for ecology and diversity studies. Sci Rep. 2016; 6.

# Disease indicators

S1 Figure. Disease indicators. Remission at baseline was defined by FC < 100 μg/g and CRP < 5 mg/L or FC < 100 μg/g, CRP < 10 mg/L, and HBI ≤ 4. Disease activity at the second visit was defined by FC, serum CRP and HBI, i.e. FC > 250 μg/g or FC > 100 μg/g with at least a 5-fold increase from baseline.

# Sensitivity analyses: Differences in abundance between healthy individuals and CD patients

S2 Figure. Results obtained with quantile regression for base case families and variables, adjusted with the Benjamini-Hochberg procedure. The red boxes are negative estimates, the green boxes are positive estimates, and the empty boxes are the variables that were not selected during variable selection. Significant variables (P-value < 0.05 after BH adjustment) are indicated with an asterisk (‘*’).

S3 Figure. Results obtained with quantile regression for base case families and variables and the RR group as reference instead of the HC group. The red boxes are negative estimates, the green boxes are positive estimates, and the empty boxes are the variables that were not selected during variable selection. Significant variables (P-value < 0.05) are indicated with an asterisk (‘*’).

S4 Figure. Violin plots of the transformed relative abundances of the selected genera per group. In blue the healthy controls, in green the RR group, and in red the RE group, all visualized per timepoint (V1 = visit 1 and V2 = visit 2). Patients in the RE group are in remission during the first visit and experience an exacerbation during the second visit. The 50% quantile is shown with a black horizontal line.

S5 Figure. Results obtained with quantile regression for the selected genera and variables. The corresponding family names are placed in bold on the left. The red boxes are negative estimates, the green boxes are positive estimates, and the empty boxes are the variables that were not selected during variable selection. Significant variables (P-value < 0.05) are indicated with an asterisk (‘*’).

S6 Figure. Results obtained with quantile regression for the selected genera and variables, adjusted with the BH procedure. The corresponding family names are placed in bold on the left. The red boxes are negative estimates, the green boxes are positive estimates, and the empty boxes are the variables that were not selected during variable selection. Significant variables (P-value < 0.05 after BH adjustment) are indicated with an asterisk (‘*’).

S7 Figure. Violin plots of the transformed relative abundances of the families outside the base case selection criterium per group. In blue the healthy controls, in green the RR group, and in red the RE group, all visualized per timepoint (V1 = visit 1 and V2 = visit 2). Patients in the RE group are in remission during the first visit and experience an exacerbation during the second visit. The 50% quantile is shown with a black horizontal line.

S8 Figure. Results obtained with quantile regression of the families outside the base case selection criterium and variables. The red boxes are negative estimates, the green boxes are positive estimates, and the empty boxes are the variables that were not selected during variable selection. Significant variables (P-value < 0.05) are indicated with an asterisk (‘*’).

S9 Figure. Results obtained with quantile regression of the families outside the base case selection criterium and variables, adjusted with the BH procedure. The red boxes are negative estimates, the green boxes are positive estimates, and the empty boxes are the variables that were not selected during variable selection. Significant variables (P-value < 0.05 after BH adjustment) are indicated with an asterisk (‘*’).

S10 Figure. Comparing the results of the LQMM analysis (20%, 50%, 80% quantiles) with the results of the linear mixed effect models. The point estimates, 95% confidence intervals and a reference line at 0 (in black) are shown. When the horizontal lines do not cross the vertical reference line, this means that the coefficients are significantly different from 0.

# Sensitivity analyses: Gut microbiota changes in relation to Crohn’s disease activity

S11 Figure. Results obtained with quantile regression for base case families for CD patients cohort data only, with correction for clinical variables and p-value adjustment according BH procedure. The red boxes are negative estimates, the green boxes are positive estimates, and the empty boxes are the variables that were not selected during variable selection. Significant variables (P-value < 0.05 after BH adjustment) are indicated with an asterisk (‘*’).

S12 Figure. Results obtained with quantile regression for the selected genera and variables for CD patients cohort data only, with correction for clinical variables. The corresponding family names are placed in bold on the left. The red boxes are negative estimates, the green boxes are positive estimates, and the empty boxes are the variables that were not selected during variable selection. Significant variables (P-value < 0.05) are indicated with an asterisk (‘*’).

S13 Figure. Results obtained with quantile regression for the selected genera and variables for CD patients cohort data only, with correction for clinical variables and p-value adjustment according BH procedure. The corresponding family names are placed in bold on the left. The red boxes are negative estimates, the green boxes are positive estimates, and the empty boxes are the variables that were not selected during variable selection. Significant variables (P-value < 0.05 after BH adjustment) are indicated with an asterisk (‘*’).

S14 Figure. Results obtained with quantile regression of the families outside the base case selection criterium for CD patients cohort data only, with correction for clinical variables. The red boxes are negative estimates, the green boxes are positive estimates, and the empty boxes are the variables that were not selected during variable selection. Significant variables (P-value < 0.05) are indicated with an asterisk (‘*’).

S15 Figure. Results obtained with quantile regression of the families outside the base case selection criterium for CD patients cohort data only, with correction for clinical variables and correction with the BH procedure. The red boxes are negative estimates, the green boxes are positive estimates, and the empty boxes are the variables that were not selected during variable selection. Significant variables (P-value < 0.05 after BH adjustment) are indicated with an asterisk (‘*’).

# Sensitivity analyses: Bacterial families relative abundances in relation to different disease activity indicators

S16 Figure. Results obtained for the different disease activity indicators with correction for clinical variables. The estimates for Status, HBI, CRP, and FC were estimated in different models, therefore the data was normalized beforehand to make the models comparable. On this purpose, the values for HBI, CRP, and FC were divided by the difference between the 5^th^ and 95^th^ percentiles. Significant variables are indicated with a closed circle.

S17 Figure. Results obtained for the different disease activity indicators with correction for clinical variables, with p-value adjustment according BH procedure. The estimates for Status, HBI, CRP, and FC were estimated in different models, therefore the data was normalized beforehand to make the models comparable. On this purpose, the values for HBI, CRP, and FC were divided by the difference between the 5^th^ and 95^th^ percentiles. Significant variables are indicated with a closed circle.

S18 Figure. Results obtained for the different disease activity indicators for the selected genera and variables for CD patients cohort data only. The estimates for Status, HBI, CRP, and FC were estimated in different models, therefore the data was normalized beforehand to make the models comparable. On this purpose, the values for HBI, CRP, and FC were divided by the difference between the 5^th^ and 95^th^ percentiles. Significant variables are indicated with a closed circle.

S19 Figure. Results obtained for the different disease activity indicators for the selected genera and variables and p-value adjustment according BH procedure for CD patients cohort data only. The estimates for Status, HBI, CRP, and FC were estimated in different models, therefore the data was normalized beforehand to make the models comparable. On this purpose, the values for HBI, CRP, and FC were divided by the difference between the 5^th^ and 95^th^ percentiles. Significant variables are indicated with a closed circle.

S20 Figure. Results obtained for the different disease activity indicators of the families outside the base case selection criterium and variables for CD patients cohort data only. The estimates for Status, HBI, CRP, and FC were estimated in different models, therefore the data was normalized beforehand to make the models comparable. On this purpose, the values for HBI, CRP, and FC were divided by the difference between the 5^th^ and 95^th^ percentiles. Significant variables are indicated with a closed circle.

S21 Figure. Results obtained for the different disease activity indicators of the families outside the base case selection criterium and variables and p-value adjustment according BH procedure for CD patients cohort data only. The estimates for Status, HBI, CRP, and FC were estimated in different models, therefore the data was normalized beforehand to make the models comparable. On this purpose, the values for HBI, CRP, and FC were divided by the difference between the 5^th^ and 95^th^ percentiles. Significant variables are indicated with a closed circle.

1. ^1^ Disease location: colonic (C), ileal (I), or ileocolonic (IC)

   ^2^ Age at diagnosis: younger than 40 years (A2) or older than 40 years (A3) [↑](#footnote-ref-1)
2. ^3^ All biological treatments concerned anti-TNF therapy

   ^4^ PPI: proton pump inhibitors

   ^5^ HBI: Harvey Bradshaw index (S1 Fig.)

   ^6^ CRP: serum C-reactive protein (S1 Fig.)

   ^7^ FC: fecal calprotectin (S1 Fig.) [↑](#footnote-ref-2)
3. ^1^  Six RR and five RE patients had a medication change between consecutive samples during the study period. In the RR group, mesalazine was stopped by one patient, prednisone by one patient and biologicals by two patients, while one patient started mesalazine and one patient started with biologicals. In the RE group, two patients started with biologicals, two patients stopped with thiopurines, and one patient started with mesalazine.

   ^2^ Ciprofloxacin and cotrimoxazole were used two and one month prior to sample collection, respectively. [↑](#footnote-ref-3)
